# Circumscription and synopsis of *Eugenia* section *Speciosae* Bünger & Mazine (Myrtaceae)

**DOI:** 10.3897/phytokeys.61.7904

**Published:** 2016-02-25

**Authors:** Mariana de Oliveira Bünger, Fiorella Fernanda Mazine, Eve J. Lucas, João Renato Stehmann

**Affiliations:** 1 Laboratório de Sistemática Vegetal, Departamento de Botânica, Instituto de Ciências Biológicas, Universidade Federal de Minas Gerais, CEP 31270-901, Belo Horizonte, MG, Brazil; 2 Departamento de Ciências Ambientais, Universidade Federal de São Carlos – campus Sorocaba, João Leme dos Santos, Km 110 - SP-264, CEP 18052-780, Sorocaba, SP, Brazil; 3 Royal Botanic Gardens, Kew, Richmond, Surrey, TW9 3AE, U.K.

**Keywords:** Amazon Forest, Atlantic Forest, Neotropics, Myrteae

## Abstract

A new section of *Eugenia* (Myrtaceae) is described, segregate from Eugenia sect. Phyllocalyx. Phylogenetic studies suggest that Eugenia sect. Phyllocalyx as traditionally delimited is paraphyletic. To maintain the monophyly of each of the sections in *Eugenia* *s.l.*, we herein opt to circumscribe a new section and recognize six taxa in sect. *Speciosae*, which has a distribution mostly in southeastern Brazil and northern South America. Nomenclatural notes are made and a taxonomic key is provided for the species of the section.

## Introduction

*Eugenia* Linnaeus is a widespread tropical genus with about 385 species in Brazil (Govaerts et al. 2014, Sobral et al. 2015), most of which grow along the Brazilian Atlantic rainforest biome (Oliveira-Filho and Fontes 2000). *Eugenia* is unique among Myrtaceae in having a native distribution that spans nearly the entire geographic range of the family (Snow 2011).

The genus *Phyllocalyx* was segregated from *Eugenia* by Otto Berg (1856) being characterized especially by peduncles with leaf-like bracts and showy sepals, proportionally larger than the flowers. The name *Phyllocalyx* O. Berg (1856) is also illegitimate, being a later homonym of *Phyllocalyx* A. Rich. (1847). When Niedenzu, in 1893, transfered *Phyllocalyx* O. Berg to *Eugenia*, he named it Eugenia sect. Phyllocalyx. This name is treated as *nomen novum* and has the same type as the illegitimate name. It has priority from 1893 and must be cited as Eugenia sect. Phyllocalyx Nied., not as Eugenia sect. Phyllocalyx (O. Berg) Nied. (McNeill et al. 2012 – Article 58.1).

Recently, based on a molecular (nuclear and plastid markers) phylogenetic analysis, Mazine et al. (2014) recognized nine clades in *Eugenia* s. l. They also confirmed the inclusion of *Calycorectes*, *Hexachlamys*, and *Phyllocalyx* in *Eugenia*. The “Phyllocalyx clade” or “clade 6” sensu Mazine et al. (2014) refers to Eugenia sect. Phyllocalyx Nied. comprising c. 15 species widely distributed in the Atlantic Forest, from eastern Brazil to Paraguay. The section is characterized by peduncles with leaf-like bracts and showy sepals, proportionally larger than the flowers (Berg 1856, under *Phyllocalyx*), and is currently being monographed (Bünger et al. unpubl. res.). A remarkable result of Mazine et al. (2014) is the placement of *Eugenia wentii* – traditionally included in Eugenia sect. Phyllocalyx (Mc Vaugh 1969) – in “clade 9” althoug this clade does not have any support.

After broad sampling of Eugenia sect. Phyllocalyx within a molecular framework (using five markers, one nuclear and four plastid) (Bünger et al. unpubl. res.), results show that Eugenia sect. Phyllocalyx sensu Berg emerges as a paraphyletic group. The clade containing most species previously placed in section *Phyllocalyx* and also containing the type-species of the section (*Eugenia involucrata* DC.) emerges as a well-supported monophyletic group (PP Bayes: 0.99; PP Beast: 0.97; ML: 75). A second, also well-supported clade (PP Bayes: 1; PP Beast: 1; ML: 100) includes species previously included in Eugenia sect. Phyllocalyx (*Eugenia bunchosiifolia* Nied., *Eugenia hermesiana* Mattos, *Eugenia longipetiolata* Mattos, *Eugenia macedoi* Mattos, *Eugenia speciosa* Cambess and *Eugenia wentii* Amshoff) but emerges with high support (PP Bayes: 0.99; PP Beast: 0.99; ML: 72) as sister to clade 9 *sensu* Mazine et al. (2014). Now, the clade 9 also emerges with high support (PP Bayes: 0.99; PP Beast: 0.99; ML: 86).

Bünger et al. (unpubl. res.) also have optimised morphological characters across the molecular tree, presenting useful results with which to distinguish the sections. Results indicated that these characters are uncommon in *Eugenia* *s.l.* and can therefore be used to support placement of species inside a genus/subgenus/section (e.g. Berg 1857, Niedenzu 1893, McVaugh 1969, Mattos 1989). Although these two clades do not emerge in a monophyletic group, they share the floral characters of showy sepals and bracteoles that could be homoplastic characters in *Eugenia* *s.l.*

To avoid continued recognition of a paraphyletic taxon we herein recognize a new section called Eugenia sect. Speciosae and provide the new circumscription of Eugenia sect. Speciosae, an identification key and a synopsis of the known species of this new section.

## Taxonomic synopsis

The section name “*Speciosae*” was chosen based on the fact that *Eugenia speciosa* is the most geographically widespread species in this group. The specific epithet “*speciosa*” is also the oldest within the section (Cambessédes 1832)

### Eugenia sect. Speciosae

Taxon classificationPlantaeMyrtalesMyrtaceae

Bünger & Mazine sect. nov.

urn:lsid:ipni.org:names:77153396-1

#### Notes.

Trees or shrubs; hairs simple. Indeterminate inflorescence which produces a floral region that, for instance, produces monads, dyads or triads and vegetative innovative shoots, as an auxotelic inflorescence (Briggs and Johnson 1989); bracteoles linear or narrowly elliptic persistent at anthesis but caducous in mature fruits; flowers showy always 4–merous; sepals showy, free, foliaceous, sepals and petals concealing the apex of the bud; ovary 2–locular; ovules 2–many, placenta axile. Fruit crowned by the calyx lobes. Seeds 1–2; seed coat membranous or cartilaginous; embryo with fused cotyledons.

#### Type.

*Eugenia speciosa* Cambess. Fl. Bras. Merid. 2 (19): 351. 1832.

Eugenia sect. Speciosae contains six species with three occurring in the Atlantic Forest of Brazil, and one distributed in northern South America, in the Amazon. The Atlantic Forest-Amazon disjunction distribution represents a classic biogeographic pattern of the Southern Hemisphere (McVaugh 1968).

### 1. Eugenia bunchosiifolia

Taxon classificationPlantaeMyrtalesMyrtaceae

Nied., Nat. Pflanzenfam. 3, Abt. 7: 82. 1893.

Eugenia bunchosiifolia Basionym: Phyllocalyx grandifolius O. Berg, Fl. Bras. 14(1): 333. 1857.
Eugenia bunchosiifolia Type: Brazil. *Habitat ad urbem Santos in prov. S.Pauli, fructificat Majo*: Sellow s.n. (holotype: B, destroyed; lectotype here designated: K[000170006]!)
Phyllocalyx grandifolius var. pyriformis O. Berg, Fl. Bras. 14(1): 591. 1859.
Eugenia bunchosiifolia Type: Brazil. *Habitat in silvis prope urbem Rio de Janeiro, e.g. ad Tejuca, florebat Novembri, fructificabat Septembri*: Riedel s.n. (holotype: LE! [photo])
Eugenia santensis Kiaerskou, Enum. Myrt. Bras. 163. 1893, nom. superfl.
Eugenia bunchosiifolia Type: Based on Phyllocalyx grandifolius O. Berg
Eugenia littoralis Mattos, Loefgrenia 42:1. 1970, nom. illeg.
Eugenia bunchosiifolia Type: Brazil. São Paulo: Peruibe, Prainha, 25 Jul 1969, Mattos 15599 (holotype: HB!)
Eugenia brunoi Mattos, Loefgrenia 99:2. 1990, syn. nov.
Eugenia bunchosiifolia Type: Based on Eugenia littoralis Mattos

#### Notes.

*Eugenia bunchosiifolia* is a tree 3–15m alt. from the coastal Atlantic Forest of Brazil, growing in rainforests from Paraná, Rio de Janeiro and São Paulo states. This species has glabrous leaves with obscure glandular dots visible on both faces, leaf apices are acuminate, without cartilaginous margins, the hypanthium is velutinous. The lectotype of *Eugenia bunchosiifolia* was chosen because the holotype was destroyed in the Second World War. The specimem found at K was a isotype and now considered the lectotype of this name.

The protologue and the examinated holotype of *Eugenia brunoi* matches with those of *Eugenia bunchosiifolia*, hence this species is here synonymized with *Eugenia bunchosiifolia*

### 2. Eugenia hermesiana

Taxon classificationPlantaeMyrtalesMyrtaceae

Mattos, Loefgrenia 94: 1. 1989

#### Type.

Brazil. São Paulo: Salesópolis, na Estação Biológica de Boracéia, 15 Jan. 1968, *Rabello, E. s/n.* (holotype: HAS, not found).

#### Notes.

This species has glabrous leaves without cartilaginous margins, dots visible mostly abaxially, leaf apices are acute or obtuse, the hypanthium is velutinous. *Eugenia hermesiana* is a shrub up to 3 m high from São Paulo State (Brazil), growing in the coastal Atlantic Forest. There are few specimens located in BHCB, IAC, NY, SP and SPSF. It is a threatened species classified as Endangered in the Brazilian Official List of Flora Threatened Species (MMA 2014).

### 3. Eugenia longipetiolata

Taxon classificationPlantaeMyrtalesMyrtaceae

Mattos, Dusenia 8: 162. 1968.

Fig. 1D

Eugenia longipetiolata Basionym: Stenocalyx mutabilis O. Berg, Fl. Bras. 14(1): 347. 1857.
Eugenia longipetiolata Type: Brazil. Tingua, Schott 5854 (lectotype here designated M [M-0170971]!; isolectotype W! [photo])
Eugenia mutabilis Nied., Nat. Pflanzenfam. 3, Abt. 7: 81. 1893, nom. illeg.
Eugenia longipetiolata Type: Based on Stenocalyx mutabilis O. Berg
Eugenia tinguana Mattos, Loefgrenia 123: 1. 2006, nom. superfl.
Eugenia longipetiolata Type: Based on Stenocalyx mutabilis O. Berg

#### Notes.

*Eugenia longipetiolata* is a tree up to 15 m high from coastal Atlantic Forest of Brazil, growing in ombrophilous forests from Rio de Janeiro and São Paulo states. This species has leaves with visible, flat gland dots on both faces, black-floccose simple trichomes on abaxial faces, caudate apices, non-cartilaginous margins and a ferruginous-pubescent hypanthium.

The lectotype was chosen for *Stenocalyx mutabilis* because Berg did not indicate a single specimen and Mattos did not designate a ectotype when he published the nom. nov.. The specimen from M was seen and here considered the lectotype for the name.

**Figure 1.**
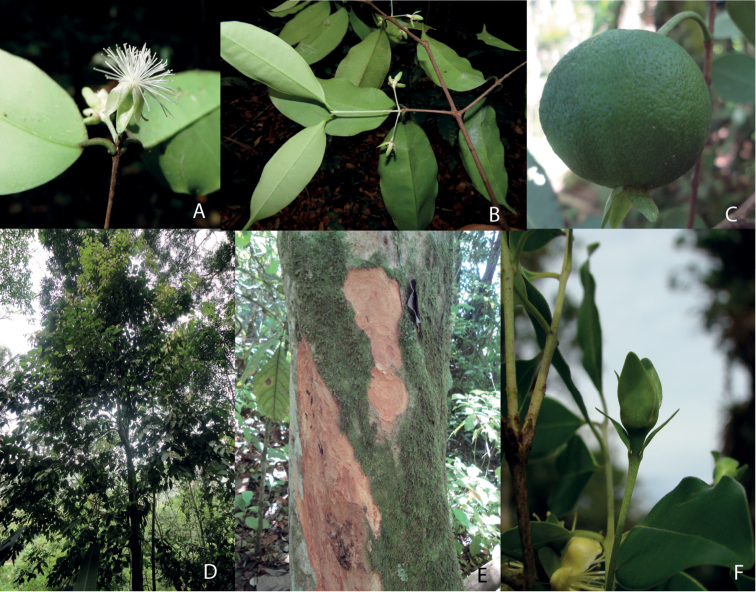
**A, B** Auxotelic inflorescence and foliaceous calyx, *Eugenia wentii* (photo by B. Holst) **C, E, F** *Eugenia speciosa*, mature fruit with decidous bracteoles, stem bark and position and format of the bracteoles (photos by M.O.Bünger); D *Eugenia longipetiolata* (photo by M.O.Bünger).

### 4. Eugenia macedoi

Taxon classificationPlantaeMyrtalesMyrtaceae

Mattos & D. Legrand, Loefgrenia 67: 24. 1975.

#### Type.

Brasil, Minas Gerais: Ituiutaba, San Vicente, 12 Sep. 1950, *Macedo, A. 2574* (holotype: MVM, not seen; isotype US! [00603977])

#### Notes.

*Eugenia macedoi* is known only by two specimens colected in Minas Gerais and Goiás States (Brazil). This species is a shrub growing in the Cerrado biome (like *savannas*). Apparently it is the only species of the section that occurs in dry areas. This species has glabrescent leaves without cartilaginous margins, dots visible on both faces, leaf apices are acute, and the hypanthium is velutinous.

### 5. Eugenia speciosa

Taxon classificationPlantaeMyrtalesMyrtaceae

Cambess., Fl. Bras. Merid. (quarto ed.) 2(19): 351. 1832.

Fig. 1C, E, F

Phyllocalyx speciosus (Cambess.) O. Berg, Fl. Bras. 27(2–3): 307. 1856.
Eugenia longipetiolata Type: Based on Eugenia speciosa Cambess.
Phyllocalyx retusus O. Berg, Fl. Bras. 14(1): 331. 1857.
Eugenia speciosa Type: Brazil. *Habitat ad ripas flaminis Rio Pardo in Montevideo*: Sellow s.n. (holotype B, probably destroyed; isotypes: K! [000276590], BR! [0000005261277]).
Phyllocalyx limbatus O. Berg, Fl. Bras. 14(1): 332. 1857.
Eugenia speciosa Type: Brazil. *Habitat ad Angra dos Reys in prov. Rio de Janeiro: Pohl 264, 5760., loco incerto ajusdem prov., Sellow* s.n. (lectotype here designated BR! [526061-Sellow]!; isolectotype: B (fl.), probably destroyed; W (fr.) [photo]!) .
Phyllocalyx macrosepalus O. Berg, Fl. Bras. 14(1): 332. 1857.
Eugenia speciosa Type: Brazil. *Habitat ad Alegres et Manoel Jesu praedia in prov. Minarum*: Mikan s.n., Pohl s.n. (lectotype here designated: BR! [526984]; isolectotypes: M! [M-0171010], W [photo]!).
Phyllocalyx marginatus O. Berg, Fl. Bras. 14(1): 332. 1857.
Eugenia speciosa Type: Brazil. *Habitat in prov. Rio de Janeiro*: Martius s.n. (holotype: BR! [526094].
Eugenia retusa (O.Berg) Nied., Nat. Pflanzenfam. 7: 82. 1893.
Eugenia longipetiolata Type: Based on Phyllocalyx retusus O. Berg
Eugenia caldensis Kiaerskou, Enum. Myrt. Bras. 162. 1893.
Eugenia longipetiolata Type: Based on Phyllocalyx marginatus O. Berg
Eugenia macrocalyx Mart. ex B.D.Jacks, Index Kew. 1: 908. 1893.
Eugenia longipetiolata Type: Based on Phyllocalyx macrosepalus Berg

#### Type.

Brazil. *In sabulosis prope praedium vulgo Fazenda d’Araucaria in prov. S.Pauli, floret Octobri*: Saint-Hilaire s.n. (lectotype: P [P01902768]!; isolectotype: MPU! [photo])

#### Notes.

*Eugenia speciosa* is a tree 5–12 m high from Atlantic Forest in southern and southeastern Brazil. It is common in rainforests and “restingas”. This species also occurs in montane Atlantic Forest in Minas Gerais State (Brazil) and also occurs in Paraguay, Argentina, Uruguay and Bolivia. This species has leaves with visible, salient dots on both faces, glabrous, obtuse apices with cartilaginous margins and a glabrous hypanthium.

The lectotypes chosen for *Phyllocalyx limbatus* and *Phyllocalyx macrosepalus* are from BR; they were seen and we consider that the specimens that well represent the names. The lectotype that was chosen for *Phyllocalyx limbatus* is a specimen that is a duplicate (isotype) of the specimen that was in B which was destroyed in the Second World War. For thus, we consider it as the lectotype for this species.

### 6. Eugenia wentii

Taxon classificationPlantaeMyrtalesMyrtaceae

Amshoff, Recueil Trav. Bot. Néerl. 39: 160, f. 4. 1942.

Fig. 1A, B

Phyllocalyx wentii Amshoff, Recueil Trav. Bot. Néerl. 39: 158, f. 4. 1942
Eugenia wentii Type: Based on Eugenia wentii Amshoff nomen alternativ.
Calycorectes macrocalyx Rusby, Mem. New York Bot. Gard. 7: 313. 1927.
Eugenia wentii Type: Bolivia. Bopi River Valley. Rusby 666 (holotype: NY! [00386736]; isotypes: BKL! [photo], MICH! [photo], US! [photo])
Eugenia macrocalyx (Rusby) McVaugh, Fieldiana, Bot. 29(3): 212. 1956, nom. illeg.
Eugenia wentii Type: Based on Calycorectes macrocalyx Rusby

#### Type.

Suriname. Fluv. Coppename inf., Went FAFC 142 (holotype: U! [0005034])

#### Notes.

*Eugenia wentii* is a treelet or tree 3–6 m high from the Amazon forest; it is found in Amazônia and Pará States (Brazil), French Guyana, Suriname, Venezuela, Bolivia, Colombia, Ecuador and Peru. This species has glabrous leaves with flat, visible gland dots on both faces, caudate apices without cartilaginous margins and a velutinous hypanthium.

### Key to species of *Eugenia* sect. *Speciosae*

**Table:** 

1	Hypanthium glabrous	***Eugenia speciosa***
–	Hypanthium with trichomes	
2	Leaves with caudate apices and black-floccose indument on mature leaves	***Eugenia longipetiolata***
–	Leaves with acuminate apices, acute, obtuse or rostrate; glabrous or without black-floccose hairs	
3	Leaves usually with cartilaginous margins	***Eugenia bunchosiifolia***
–	Leaves always without cartilaginous margins	
4	Leaves with acuminate or rostrate apices	***Eugenia wentii***
–	Leaves with acute or obtuse apices	
5	Calyx lobes acuminate 50 to 70 mm long.	***Eugenia hermesiana***
–	Calyx lobes acute 3.9 to 7 mm long.	***Eugenia macedoi***

## Supplementary Material

XML Treatment for Eugenia sect. Speciosae

XML Treatment for Eugenia bunchosiifolia

XML Treatment for Eugenia hermesiana

XML Treatment for Eugenia longipetiolata

XML Treatment for Eugenia macedoi

XML Treatment for Eugenia speciosa

XML Treatment for Eugenia wentii
